# AGE MODERATES THE LINK BETWEEN DAILY POSITIVE EVENTS AND WELL-BEING: A META-ANALYSIS

**DOI:** 10.1093/geroni/igac059.481

**Published:** 2022-12-20

**Authors:** Patrick Klaiber, Lydia Ong, Nancy Sin

**Affiliations:** University of British Columbia, Department of Psychology, Vancouver, British Columbia, Canada; University of British Columbia, Vancouver, British Columbia, Canada; University of British Columbia, Vancouver, British Columbia, Canada

## Abstract

Daily positive events are linked to better health and well-being, and this association might be stronger for older adults due to age-related changes in motivations and emotion regulation. We therefore investigated whether age moderated the links between positive events and health-related outcomes, using data from a meta-analysis of 142 studies spanning 50 years of research. Multilevel meta-analytical moderation analyses revealed stronger correlations of positive events with better well-being (i.e. higher positive affect and life satisfaction), mental health, and lower disability in older compared to younger samples (p’s < .05). No age moderation was found for links of positive events with social connection, health behaviors, physical symptoms, perceived stress, self-reported health, and inflammation. Findings suggest that although daily positive events were protective for health and well-being for all adult age groups, these experiences may be especially beneficial for positive well-being and mental health in late life.

